# Cuproptosis-related gene *CEP55* as a biomarker of pancreatic adenocarcinoma via multi-omics techniques and experimental validation

**DOI:** 10.2478/raon-2025-0042

**Published:** 2025-07-18

**Authors:** Riyuan Zhang, Zixia Xu, Yurui Zhuang, Yuzhe Shi, Ziyi Guo, Chong Chen

**Affiliations:** Department of Hepatobiliary and Pancreatic Surgery, People’s Hospital of Pingyang County, Wenzhou, China; The First Clinical Medical College, Wenzhou Medical University, Wenzhou, China; Departments of Endocrinology, Minhang Hospital, Fudan University, Shanghai, China

**Keywords:** cuproptosis, pancreatic adenocarcinoma, multi-omics techniques, single-cell analysis

## Abstract

**Background:**

Background. Pancreatic adenocarcinoma (PAAD) is a malignancy with a very poor prognosis. The clinical significance of cuproptosis in PAAD combining single cell data with The Cancer Genome Atlas (TCGA) data is unclear.

**Materials and methods:**

In this study, we first identified gene modules associated with cuproptosis by performing single-cell analysis and weighted co-expression network analysis (WCGNA). According to TCGA data, Cox regression and LASSO regression analysis were used to establish prognostic models, and PAAD patients were divided into high-risk and low-risk groups according to cuproptosis-related risk score. Then 7 algorithms were used to evaluate cancer immune microenvironment, followed by the mutation analysis. The expression levels and prognostic significance of the 8 model genes were analysed using single-gene analysis, Kaplan-Meier survival plots, and quantitative PCR (qPCR) validation. Finally, the biological function of *CEP55* in PAAD was verified by in vitro experiments.

**Results:**

We identified cuproptosis-related genes (CRG) in PAAD by performing single-cell analysis and WCGNA, and constructed a cuproptosis-related prognostic model of PAAD by comprehensive bioinformatics analyses. Based on cuproptosis-related risk score, there were significant differences in survival time between two groups. We further constructed a cuproptosis-related risk score-based nomogram to accurately assess PAAD patient prognosis. Immune infiltration analysis revealed that PAAD samples with higher cuproptosis-related scores exhibited significantly lower immune infiltration levels, which may mechanistically underlie their poorer clinical outcomes. Furthermore, the high-risk group had a higher mutation rate of the same mutated gene, which means that they are more likely to benefit from immunotherapy. Finally, we identified that *CEP55* was significantly overexpressed in PAAD and correlated with poor patient prognosis. In vitro knockdown of *CEP55* effectively suppressed proliferation and invasion capabilities in pancreatic cancer cell lines.

**Conclusions:**

In this study, a novel prognostic model of PAAD was constructed to evaluate the prognosis and immune microenvironment of PAAD patients, and *CEP55* was identified as a central gene of PAAD. In vitro studies verified the biological function of *CEP55*, providing a new potential target for the treatment of PAAD.

## Introduction

Pancreatic adenocarcinoma (PAAD) is a fatal disease.^1–3^ Due to the deep location of the pancreas in the body and the lack of noticeable early symptoms, detection and diagnosis of PAAD has been difficult^2^ and treatment has been limited.^4^ It is estimated that more than 80% of PAAD patients are already unsuitable for surgery and have distant metastases at the time of diagnosis, for which current treatment strategies often have little effect.^5^ Recently, immunotherapies, such as PD-1/PDL-1 or CTLA-4 inhibitors, are being extensively studied for their utility in major high mutation-load solid tumors.^6^ In addition, many studies have been reported on the immune microenvironment (IME) of PAAD and the relationship between its stromal cells and immune cells.^7,8^ However, there are still some gaps in key targets, inhibitors and improved clinical prediction models of PAAD.^7^

Dysregulation of apoptosis is an essential hallmark of cancer.^9^ Therefore, reconstructing the cell death program and inducing cancer cell death have been a promising development in the field of cancer therapy.^10^ In addition to the mainstream programmed cell death, death receptor-mediated apoptosis, ferroptosis^11,12^, pyroptosis^13^, parthana-tos^14^, as well as the newly revealed cuproptosis^15^ were also involved. It was shown that as a non-ap-optotic cell death pathway, copper can bind to and aggregate with lipoylated TCA cell cycle proteins, which then trigger proteotoxic stress as well as loss of Fe-S cluster proteins, leading to cell death.^16^ Given the close link between necroptosis and immuno-oncology^17^, the role of this novel cuproptosis model is also being explored in different cancers, such as hepatocellular carcinoma^18^, breast cancer^19^ and renal clear cell carcinoma^20^, in the context of the rise of immune checkpoint therapy^20^, among others. Metabolic recombination is one of the typical characteristics of PAAD, so cuproptosis-related genes (CRGs) may provide new prognostic markers and guide the development of new therapeutic regimens.^21^

In this study, we used single-cell data to screen for differential genes associated with cuproptosis, and the data of 177 PAAD samples in TCGA were screened by the WGCNA algorithm to identify gene modules associated with cuproptosis. Subsequently, through comprehensive bioinformatics analysis, we constructed a cuproptosis-related prognostic model for PAAD and classified PAAD patients into high-risk and low-risk groups based on cuproptosis-related risk scores. An evaluation of the cancer IME using seven methods and mutational analysis revealed the mutation types in the high and low risk groups. Further analysis revealed that *CEP55* was significantly high expressed in PAAD and correlated with poor patient prognosis. Finally, we performed the in vitro study to reveal the biological function of *CEP55* in PAAD.

## Materials and methods

### Transcriptome data download and processing

We used “*TCGAbiolinks*” R package to download The Cancer Genome Atlas (TCGA) data as the training cohort. 177 transcriptomic data samples of in solid cancer with complete clinical data were obtained after eliminating non-primary tumour samples. Subsequently, we downloaded the PAAD dataset GSE85916 from the GEO database as the validation cohort, and all data were log2-transformed to be used for subsequent analysis.

### Single cell sequencing data download and processing

The single cell dataset GSE212966 for PAAD was downloaded from the GEO database. The dataset contains a total of 12 samples. The “Seurat” R package was used to analyze the single cell data. We pre-processed these data using the following standards: cells with less than 10% of mitochondrial genes, cells with the total number of genes >200 and genes with expression range of 200-7000 and being expressed in at least three cells were retained. The data normalization was performed using the *LogNormalize* method with a standard scale factor of 10,000. Subsequently, the top 2,000 most variable features were identified and selected through the *FindVariableFeatures* function. To account for mitochondrial content variation, the dataset was scaled using the *ScaleData* function with mitochondrial percentage as a key parameter. For dimensionality reduction and visualization, the Uniform Manifold Approximation and Projection (UMAP) technique was implemented to generate two-dimensional representations of the clustering results. Cluster-specific marker genes were identified using the FindAllMarkers function with stringent statistical thresholds. Finally, cell type annotation was conducted by cross-referencing the identified marker genes with well-established cell-type-specific markers documented in the literature.

### Acquisition of cuproptosis-related genes (CRGs genes)

All CRGs genes were obtained from the study of Tsvetkov P *et al*. in the journal Science.^15^ No additional ethical approval is required, as the data are available online and have usage allowance. The percentage of CRGs genes in each cell was then obtained by entering 10 CRGs genes using the PercentageFeatureSet function.

### Weighted Co-Expression Network Analysis (WGCNA)

Weighted Co-Expression Network Analysis is a systematic biological method for characterizing patterns of correlation between genes in microarray samples.^22^ This method can be used to find clusters (modules) of highly related genes. In this study, we used the WGCNA method to find gene modules in PAAD that were highly correlated with cuproptosis to obtain CRGs.

### Prognostic modeling associated with cuproptosis

A prognostic model containing 8 genes was constructed using Cox-lasso’s algorithm.^23^ Univariate Cox analysis was then conducted to screen for important key genes. Using the “glmnet” tool in the R package, LASSO Cox regression analysis is undertaken to perform further screening and construct prognostic models. After this, the CRGs scores were calculated using the formula, and patients in the TCGA PAAD cohort were divided into high-risk and low-risk groups based on the median, to explore the differences in prognosis between the two groups. Finally, we evaluated the accuracy of the model by receiver operating characteristic (ROC) analysis and principal components analysis (PCA).

### Drug sensitivity analysis

Immunotherapy sensitivity scores for the TCGA-PAAD (The Cancer Genome Atlas Pancreatic Adenocarcinoma) cohort were obtained from The Cancer Immunome Atlas (TCIA; https://www.tcia.at/home). TCIA represents a comprehensive resource that integrates next-generation sequencing (NGS) data from TCGA and other sources, providing immunogenomic profiles for 20 solid tumor types. To evaluate differential immunotherapy responses between risk groups, we employed the “ggpubr” R package to perform comparative analyses based on CRG related scores. Additionally, drug sensitivity was assessed using the “pRRophetic” package, which estimates the Half Maximal Inhibitory Concentration (IC50) for various therapeutic compounds. Lower IC50 values indicate greater drug sensitivity in patients.

### External validation of the model

GSE85916 in GEO was used as an external validation cohort. In this validation cohort, the CRGs score was calculated for each sample according to the formula of the model, and patients were divided into a high-risk group and a low-risk group based on the median of the scores. Survival analysis was performed to judge whether there was a difference in prognosis between these two subgroups. Next, we evaluated the stability of the model using ROC curves. PCA was used to explore whether the model could better group patients with PAAD.

### Correlation analysis of immune infiltration and mutation

We evaluated the IME of PAAD in high and low risk groups using seven algorithms including CIBERSORT, EPIC, Estimate, MCP_counter, Quanti-seq, TIMER, xCell and showed their results in the form of heat maps. We then performed intergroup mutation analysis and further analysis based on the results.

### Construction of Nomogram

The prognostic nomogram was developed by integrating CRGs-related risk scores, with continuous variables normalized and categorical variables incorporated using appropriate reference levels. Model coefficients were transformed into a 0-100 point scoring system using the R package ‘rms’ to generate a clinically applicable visual predictive tool. The nomogram’s discriminative performance was rigorously assessed using ROC curve analysis at 1-, 3-, and 5-year follow-up intervals, with the area under the curve (AUC) and 95% confidence intervals calculated through 1000 bootstrap iterations to evaluate predictive accuracy for survival outcomes. To further validate the model’s clinical utility, decision curve analysis (DCA) was performed across a comprehensive range of threshold probabilities (0-100%), systematically comparing the net benefit of the nomogram against default “treat-all” and “treat-none” strategies while accounting for the clinical consequences of falsepositive and false-negative predictions.

### Single gene analysis of 8 model genes

In this study, we conducted a meticulous examination of the expression profiles of the 8 model genes at the single-cell level. This involved a comprehensive exploration of their individual expression patterns across diverse cellular contexts. Subsequently, to glean insights into their clinical relevance, we performed a rigorous single-gene prognostic analysis. This investigative approach allowed us to unravel the nuanced intricacies of each gene’s expression within individual cells and, importantly, assess their potential impact on patient prognosis.

### Immune checkpoint analysis

The expression profile data from the TCGA-PAAD cohort was used to analyze the expressed levels of 79 common immune checkpoint-related genes between different CRGs-related risk scores, including *ADORA2A, BTLA, BTN2A1, BTNL3*, and *CD27*. The “ggplot2” package was utilized to generate the boxplot and we only demonstrated differentially expressed immune checkpoint related genes.

### Cell culture and real-time quantitative PCR (qPCR) validation of screened genes

HPDE6-C7 and PAAD cell lines (ASPC1 and BXPC3) were purchased from ATCC (Manassas, USA). DMEM basal medium supplemented with 10% fetal bovine serum (Gbico), 100 μg/ml penicillin and 100 mg/ml streptomycin were used for culture, respectively. All cells were incubated in an incubator with a constant temperature of 5% CO_2_ at 37°C. Experiments were performed in six independent biological replicates (cultures derived from separate passages). RNA Extraction Kit (beyotime) was used to extract the whole RNA from pancreatic epithelial cells (HPDE6-C7) and pancreatic cancer cells (ASPC1 and BXPC3), and then reverse transcribed into cDNA. SYBR green method was used for RT-qPCR. GAPDH can be used as a reference for comparing the mRNA expression levels of corresponding genes.

The sequence of RNA primers is shown below:

GAPDH: Forward: 5’-GGAGCGAGATCCCTCCAAAAT-3’,
Reverse: 5’-GGCTGTTGTCATACTTCTCATGG-3’;
CEP55: Forward: 5’-AGTAAGTGGGGATCGAAGCCT-3’,
Reverse: 5’-CTCAAGGACTCGAATTTTCTCCA-3’;
KIF23: Forward: 5’-CCATAAAACCCAAACCTCCACA-3’,
Reverse: 5’-CTATGGGAACGGCTGGACTC-3’;
ARNTL2: Forward: 5’-ACTTGGTGCTGGTAGTATTGGA-3’,
Reverse: 5’-TGTTGGACTCGAATCATCAAGG-3’;
FAM111B: Forward: 5’-GCTAGCATGAATAGCATGAAGACA-3’,
Reverse: 5’-GGATCCGCACTCCATAGG-3’;
MRPL3: Forward: 5’-TGCTGCAATTAAACCAGGCAC-3’,
Reverse: 5’-CGTTTGACCATGCGTAGCAG-3’;
DHX30: Forward: 5’-CCAGCCTCGTGATGAGGAAT-3’,
Reverse: 5’-GCTGGGCCCGATCTTTTCT-3’;
MET: Forward: 5’-TGGGCACCGAAAGATAAACCT-3’,
Reverse: 5’-CACTCCCCATTGCTCCTCTG-3’;
KNSTRN: Forward: 5’-AGGGCCTTGATCCAGCTTTA-3’,
Reverse: 5’-TACCTTTAAGGCCTGTAACTCC-3’;

### RNA interference and cell transfection

siRNAs targeting *CEP55* (si: 5’-GGACTTTTAGCAAAGATCTTT-3’) were constructed by RiboBio (Guangzhou, China). For the transient transfection process, we utilized Lipofectamine RNAiMAX reagent from Thermo Fisher Scientific (Massachusetts, USA). Briefly, cells were seeded at an appropriate density and allowed to adhere overnight in complete medium. Dilute 2μg of siRNA to be transfected and 25μl of Lipofectamine RNAiMAX reagent in 100 μl serumfree medium and mix well. Functional assays were conducted 24 hours after transfection to evaluate the impact of *CEP55* knockdown.

### Cell Counting Kit-8 (CCK-8)

In this study, we evaluated cell proliferation and viability using the ASPC1 cell line with the CCK-8 (Cell Counting Kit-8) assay. Initially, ASPC1 cells were seeded in a 96-well plate at a density of 1×10^4 cells/well and incubated at 37°C with 5% CO_2_ for 24 hours to allow for attachment. Following this, varying concentrations of the test compounds were added, and the cells were incubated for an additional 24, 48, and 72 hours. Each condition was tested with six independent biological replicates. At the end of each treatment period, 10 μL of CCK-8 reagent was added to each well, and the plate was further incubated for 1 to 4 hours to enable the viable cells to reduce the reagent to a soluble orange formazan product. The optical density (OD) values were measured at a wavelength of 450 nm.

### Transwell

Transwell assay was performed to evaluate the migratory and invasive properties of ASPC1 cells under distinct experimental conditions: *CEP55*-knockdown (siRNA), negative control (si-NC), and untreated control (Con) groups. The assay was performed using a Transwell chamber with a polycarbonate membrane (8 μm pore size). First, ASPC1 cells were trypsinized and resuspended in serumfree medium, then seeded into the upper chamber at a density of 1×10^5 cells/well. The lower chamber was filled with a complete medium containing 10% fetal bovine serum (FBS) to create a chemotactic gradient. After 24 hours of incubation at 37°C with 5% CO_2_, non-migrated cells on the upper side of the membrane were gently wiped off with a cotton swab, while migrated cells on the lower side were fixed with 4% paraformaldehyde and stained with crystal violet. The number of migrated cells was quantified under a light microscope by randomly selecting five fields of view per membrane and counting the stained cells.

### Statistics analysis

In this study, the R software (Institute of Statistics and Mathematics, Vienna, Austria; version 4.1.2) was applied to all statistical analysis procedures. Quantitative data are expressed as mean ± SEM (standard error of the mean). Normality and homogeneity of variance were assessed using Shapiro-Wilk and Levene’s tests, respectively. For comparisons between two groups, we applied: (1) Student’s t-test for normally distributed data with equal variances; (2) Welch’s t-test for normally distributed data with unequal variances; or (3) the Wilcoxon rank-sum test (Mann-Whitney U test) for non-normally distributed data. For multiple group comparisons, we employed: (1) one-way ANOVA (with Tukey’s post hoc test) for normally distributed data with equal variances; (2) Welch’s ANOVA (with Games-Howell post hoc test) for normally distributed data with unequal variances; or (3) the Kruskal-Wallis test (with Dunn’s post hoc test) for non-normally distributed data. Each group contains 6 samples. It was statistically significant only when two-sided p value < 0.05.

## Results

Our workflow diagram is shown in Supplementary Figure 1.

**FIGURE 1.**
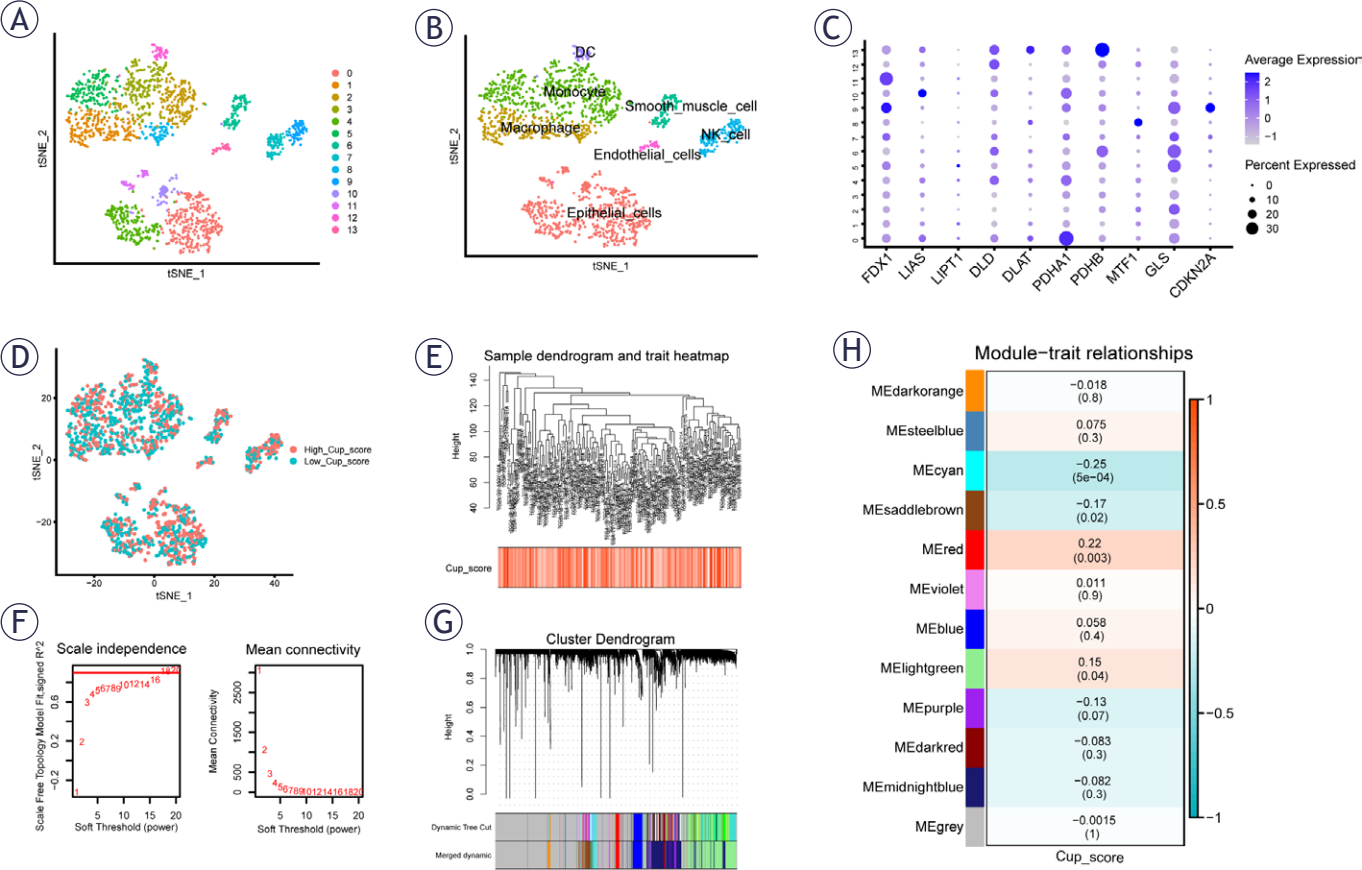
Single cell sequencing analysis of GSE212966. **(A)** Dimensionality reduction and cluster analysis. All cells in 12 samples were clustered into 14 clusters. **(B)** According to the surface marker genes of different cell types, the cells are annotated as D cells, endothelial cells, monocyte and macrophages, smooth muscle cells, natural killer (NK) cells and epithelial cells, respectively. **(C)** The expressed levels of ten cuproptosis-related genes in each cluster. **(D)** The percentage of necroptosis genes in each cell. The cells were divided into high-and low-cuproptosis cells. **(E-H)** The Weighted Co-Expression Network Analysis (WGCNA) algorithm identified gene modules associated with cuproptosis. Notably, MEred and MEcyan modules demonstrated a significant correlation with cuproptosis scores.

### Single cell sequencing data analysis

In the initial step, we integrated and scrutinized the PAAD single-cell sequencing dataset obtained from GEO. The quality control process of the single-cell analysis is depicted in Supplementary Figure 2. Illustrated in Supplementary Figure 2, these samples exhibited seamless integration without any discernible interval effects, paving the way for subsequent analysis. Utilizing the k-Nearest Neighbor (KNN) clustering algorithm, we categorized all cells into 14 clusters (Figure 1A). Additionally, seven distinct cell types were identified as dendritic cells (DC), monocyte, macrophage, endothelial cells, smooth muscle cells, natural killer (NK) cells, and epithelial cells (Figure 1B). Figure 1C illustrates the distribution of 10 CRGs genes within each cluster.

**FIGURE 2.**
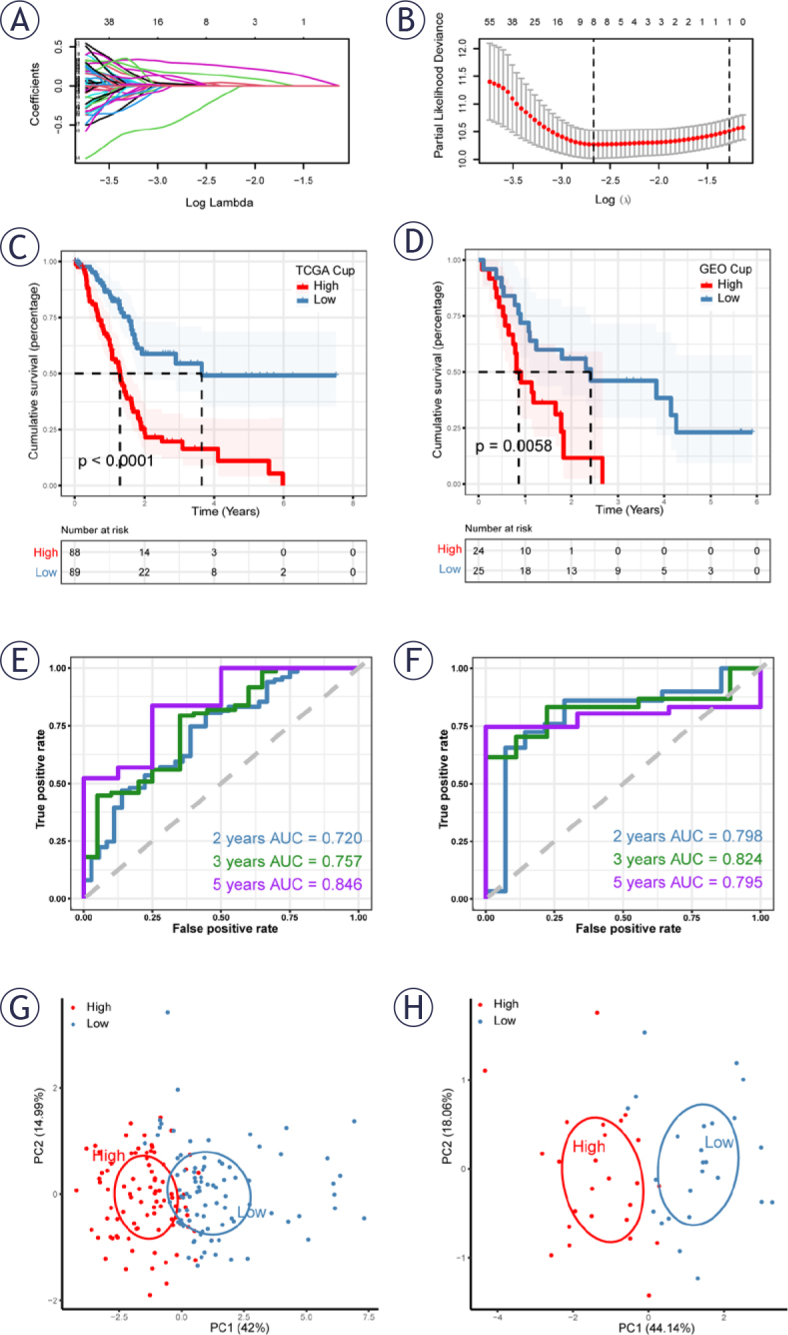
Construction and validation of cuproptosis-related prognostic model. **(A, B)** LASSO regression identified eight genes for the prognostic model construction. **(C)** The Cancer Genome Atlas (TCGA) cohort survival analysis revealed poorer prognosis in the high cuproptosis-related genes (CRGs) group (P<0.0001). **(D)** GSE85916 Cohort survival analysis indicated a worse prognosis in the high-CRGs group (P=0.0058). **(E)** ROC curve of TCGA cohort. The AUC values of the model in 2, 3 and 5 years were 0.720, 0.757 and 0.846, respectively. **(F)** ROC curve of GSE85916 Cohort. The AUC values of the model in 2, 3 and 5 years were 0.798, 0.824 and 0.795, respectively. **(G, H)** Principal components analysis (PCA) analysis in TCGA and GSE85916 cohorts demonstrated effective patient grouping in both training and validation sets.

Cells were stratified into those exhibiting low and high expression related to cuproptosis, based on the median percentage of CRGs, as illustrated in the tSNE plot (Figure 1D). The high CRGs and low CRGs groups were subjected to differentially expressed analysis, leading to the identification of a specific gene.

### Weighted Co-Expression Network Analysis WGCNA

In the TCGA cohort, cuproptosis-related score (Cup_score) was calculated by ssGSEA. Then, Cup_score related module were derived through WGCNA analysis of the transcriptome data from 177 pancreatic adenocarcinoma patients (Figure 1E–H). Employing a soft threshold of 16, a minimum module gene counts of 100, a deep Split value of 2, and merging modules with a similarity threshold below 0.5 resulted in a total of 11 nongrey modules (Figure 1F–G). Notably, MEred and MEcyan modules demonstrated a significant association with cuproptosis scores within the nongrey modules, as illustrated in Figure 1H.

Genes meeting a stringent p-value threshold of < 0.05 were selected from these three modules for subsequent analysis. Furthermore, differential expression analysis, followed by enrichment analysis of PAAD data from TCGA, revealed the pivotal involvement of immune-related processes. Key pathways such as the chemokine signalling pathway, cytokine-cytokine receptor interaction, and B cell receptor signalling pathway were identified as playing crucial roles in pancreatic adenocarcinoma, as depicted in Supplementary Figure 3.

**FIGURE 3.**
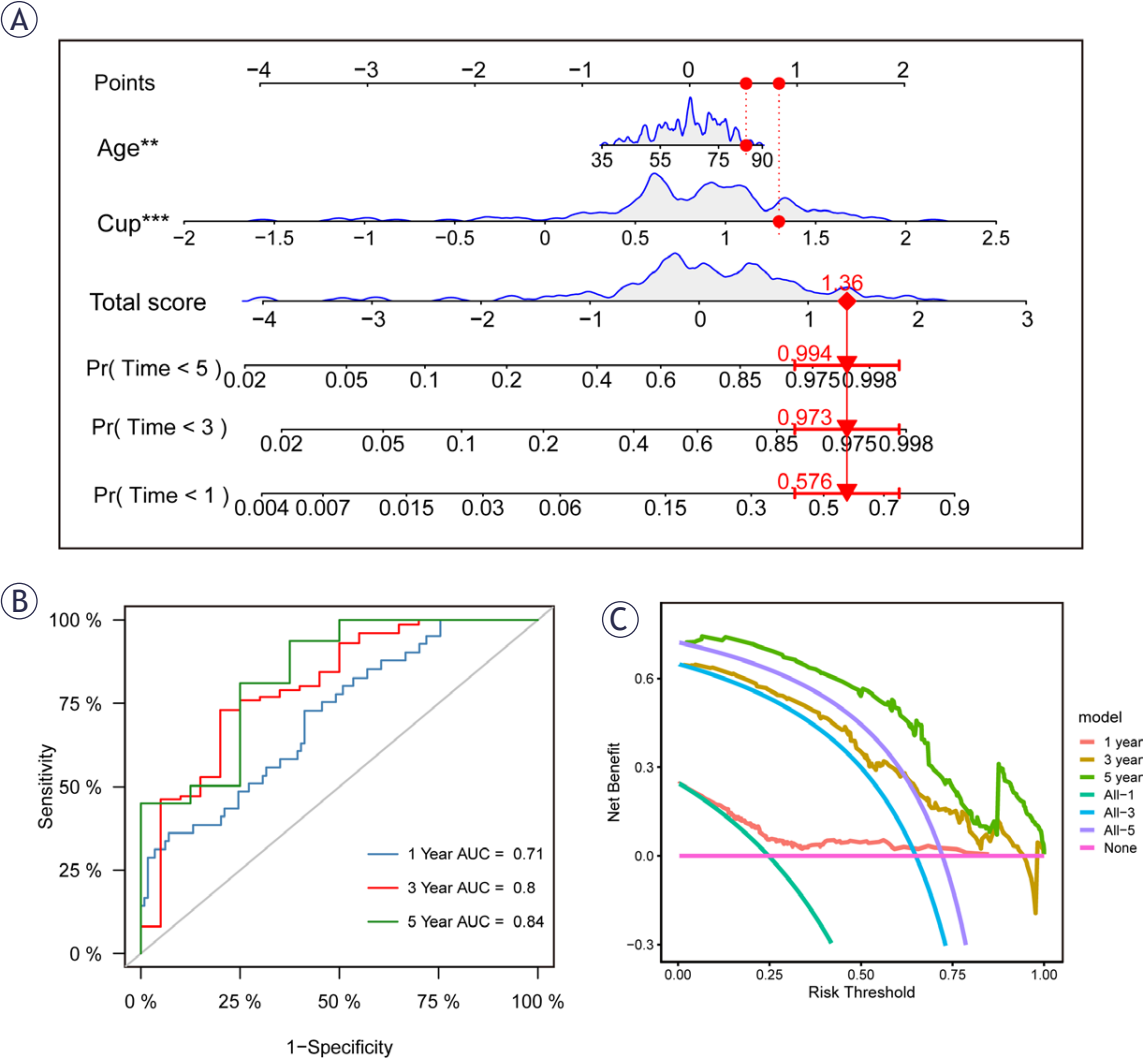
The construction of a nomogram. **(A)** Nomogram for the patient predicting mortality rates at 1, 3 and 5 years: 0.576, 0.973, and 0.994, respectively. **(B)** Nomogram ROC curve indicating AUC values at 1, 3 and 5 years as 0.71, 0.8, and 0.84. **(C)** Decision curve analysis demonstrated superior performance of the nomogram over other clinical indicators.

### Construction and validation of a cuproptosis-related prognostic model

By intersecting differentially expressed genes identified through single-cell sequencing data analysis with CRGs obtained from WGCNA, a total of 773 genes were curated. Initial selection of genes associated with patient prognosis was performed through univariate COX analysis, with a significance threshold set at P < 0.05. Subsequently, in LASSO regression analysis, gene contraction exhibited optimal stability with minimal partial likelihood bias when eight genes were included (Figure 2A–B).

The final prognostic model comprised 8 genes (*CEP55, KIF23, ARNTL2, FAM111B, MRPL3, MET, KNSTRN, DHX30*). Figure 2C illustrates a poorer prognosis in the high CRGs group within the TCGA training cohort (P < 0.0001). Similarly, in the GSE85916 validation cohort, patients with high CRGs demonstrated a significantly worse prognosis than those with low CRGs (P = 0.0058, Figure 2D).

To assess the stability of CRGs in prognostic evaluation for PAAD patients, ROC curve analysis was performed in both the training and validation cohorts. Shown in Figure 2E are ROC curves for 2-year, 3-year, and 5-year prognoses in the TCGA and GSE85916 cohorts, respectively. In the TCGA cohort, the area under the curve (AUC) values were 0.720, 0.757, and 0.846 at 2, 3, and 5 years, while in the validation cohort, the AUCs were 0.798, 0.824, and 0.795 at 2, 3, and 5 years (Figure 2F). These results affirm that CRGs exhibits high accuracy in predicting patient prognosis in both cohorts.

Finally, PCA analysis of the eight genes in the model, performed in the training and validation sets, respectively, revealed the model’s efficacy in effectively distinguishing PAAD patients into different groups (Figure 2G–H).

### The construction of a Nomogram

In order to accurately calculate the prognosis of PAAD patients, we constructed a Nomogram by cuproptosis-related scores. As the nomogram exhibited in Figure 3A, the mortality rates of patients at 1, 3, and 5 years were estimated to be 0.576, 0.973, and 0.994 according to their age and CRGs score. In essence, the column line plot is a visualization of the regression equation results that can be used to easily calculate the prognosis of PAAD patients and guide subsequent clinical decisions. To further evaluate the accuracy of this line plot, a ROC analysis was performed. The results showed that the area under the curve (AUC) was 0.71, 0.8, and 0.84 at 1, 3, and 5 years, respectively (Figure 3B). In order to evaluate the clinical utility of CRGs scores, we conducted decision curve analysis on this nomogram, and the results showed that they were above the reference line for a large threshold range, which had a good guiding effect (Figure 3C).

### Immuno-infiltration analysis and mutation environment

**FIGURE 4.**
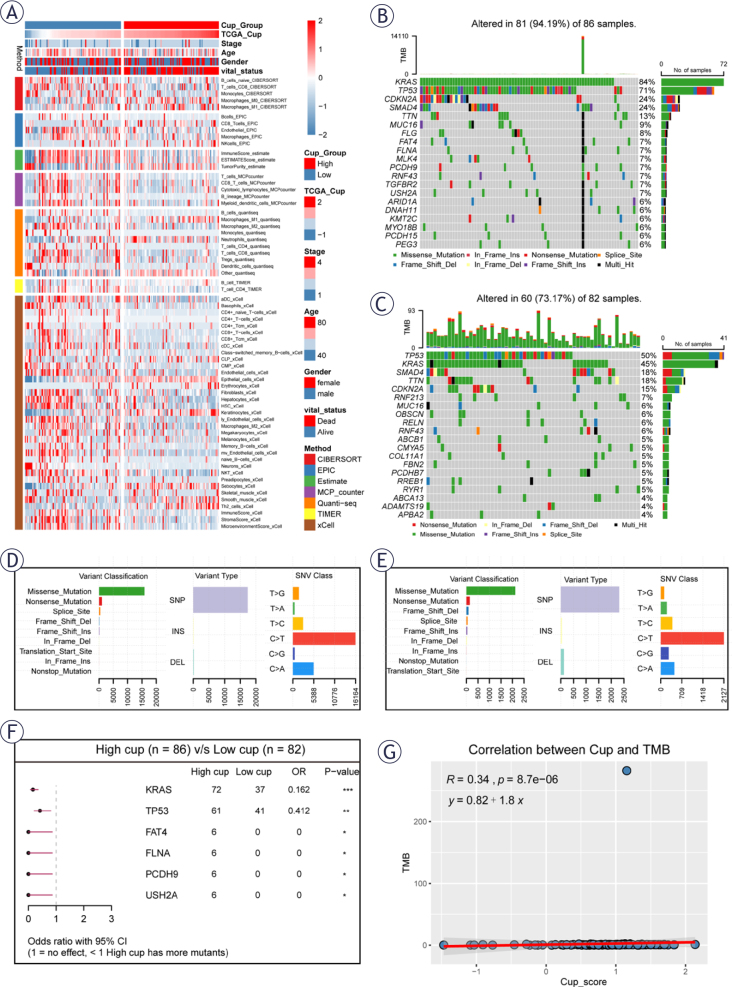
Immune microenvironment and mutation correlation analysis. **(A)** Heatmap depicts immune cell infiltration in high and low cuproptosis-related genes (CRGs) groups, with seven methods employed to assess the cancer immune microenvironment in corresponding risk groups. **(B-E)** The results of the mutation types in high-and low-CRGs groups. **(F)** Further analysis revealed that there were variations in the mutation rates of the same genes in high-and low-CRGs groups. **(G)** The CRGs risk level positive correlates with tumor mutational burden.

Through our comprehensive analysis, we’ve identified significant variations in patient outcomes within the CRGs subgroup. To unravel the etiology and guide immunotherapy, we utilized seven methods to explore differences in the cancer immune microenvironment. Results in Figure 4A depict a higher presence of immune cell infiltrates, predominantly T cells, B cells, and macrophages, in the low CRGs group. Statistical outcomes from 7 immune infiltration algorithms (Supplementary Figure 4) support these findings. Additionally, the expressed levels of immune checkpoint-related genes showed in Supplementary Figure 5A–B. We found that most of the immune checkpoint-related genes were high expressed in low CRGs group, such as *BTLA, CD160, CTLA4, LAG3, PDCD1, TIGIT, CD27, CD28*, and *TNFSRF14*.

**FIGURE 5.**
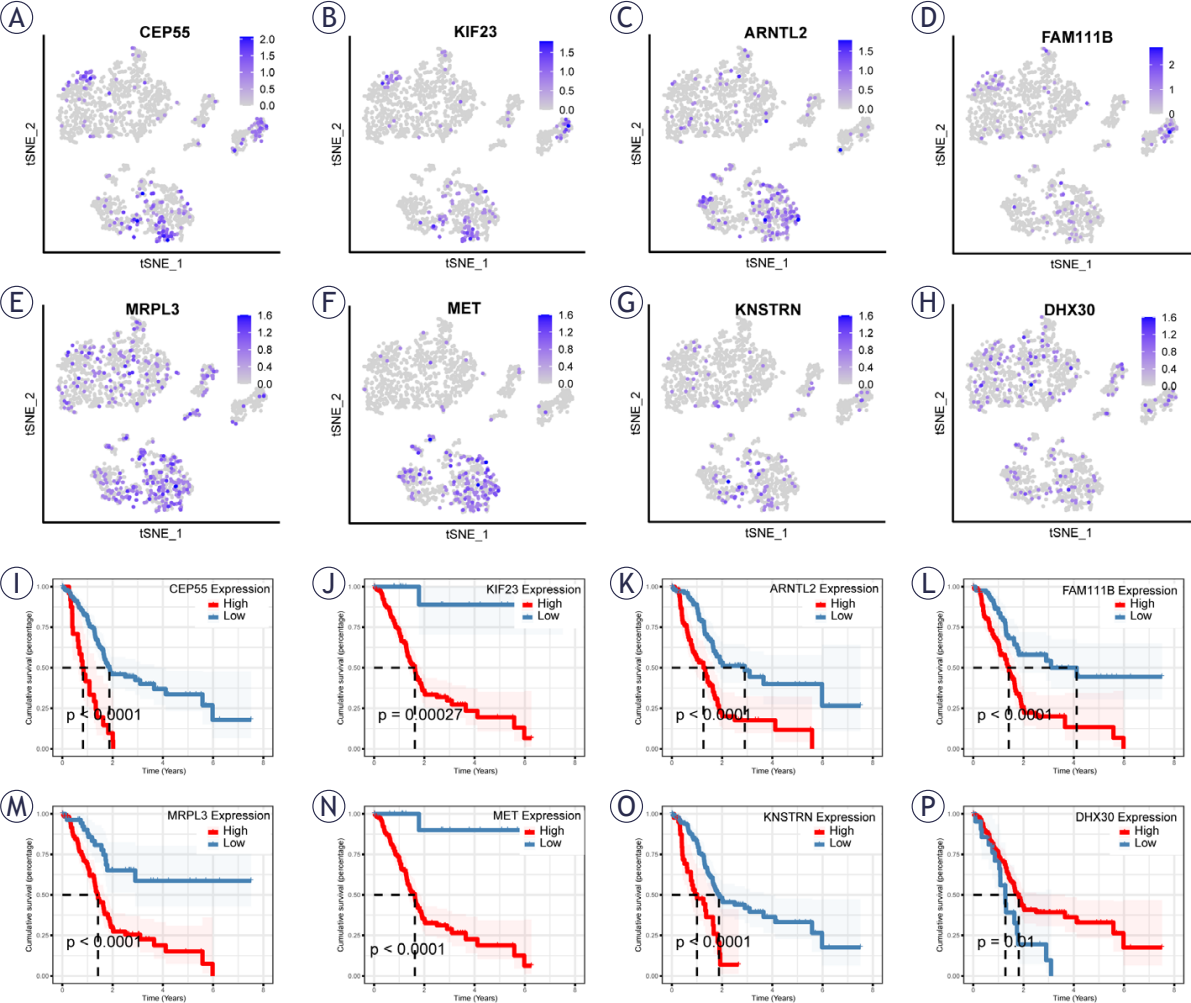
Single-cell sequencing analysis to investigate the cellular localization of 8 model genes. **(A–H)** Expression patterns of 8 genes in single cells. **(I–P)** Univariate cox analysis of the prognostic value of 8 genes.

Moving to genomic analysis, we examined the top 20 mutated genes in high and low CRGs groups. Mutation rates in the high and low CRGs group were tested separately. The results showed that *KRAS, TP53, CDKN2A, SMAD4* and *TTN* had the highest mutation rates in high and low CRGs groups and the mutation rate was higher in the high CRGs group (Figure 4B,C). Although the mutation types were similar, there were a small number of Deletion (DEL) mutations in the low CRGs group in addition to Single-nucleotide polymorphisms (SNPs) (Figure 4D,E). In addition, we also performed mutation gene association analysis in the high and low CRGs group. The results showed that *TP53* and *KARS* mutations occurred simultaneously in the high CRGs group, while *SMAD4* and *CDKN2A* mutations occurred in addition to *TP53* and *KARS* mutations in the low CRGs group. (Supplementary Figure 5C–D). Notably, a higher mutation burden was observed in the high CRGs group (Figure 4F). Correlation analysis revealed a direct relationship between CRGs (Cup) and Tumor Mutational Burden (TMB) (Figure 4G), confirming mutual validation. Our pathway analysis (Supplementary Figure 5E–F) concentrated mutations in key pathways. The results showed that *RTK-RAS* and *TP53* were highly correlated with mutation genes in high CRGs group, while *TGFBeta* and *TP53* were highly correlated with mutation genes in low CRGs group. These findings contribute to a nuanced understanding of immune microenvironment variations and genomic landscapes within distinct CRGs subgroups, offering valuable insights for cancer immunotherapy.

### Drug sensitivity intergroup differences

In order to further explore the different emphases of drug therapy for patients in high and low risk groups, 5 drugs associated with the copper ion metabolism or cuproptosis were selected for drug sensitivity analysis (Supplementary Figure 6). The results showed that Elesclomol had significant differences between high and low CRGs expression groups (Supplementary Figure 6A), and the IC50 of high risk group was lower, so patients with high CRG score were more sensitive to Elesclomol.

**FIGURE 6.**
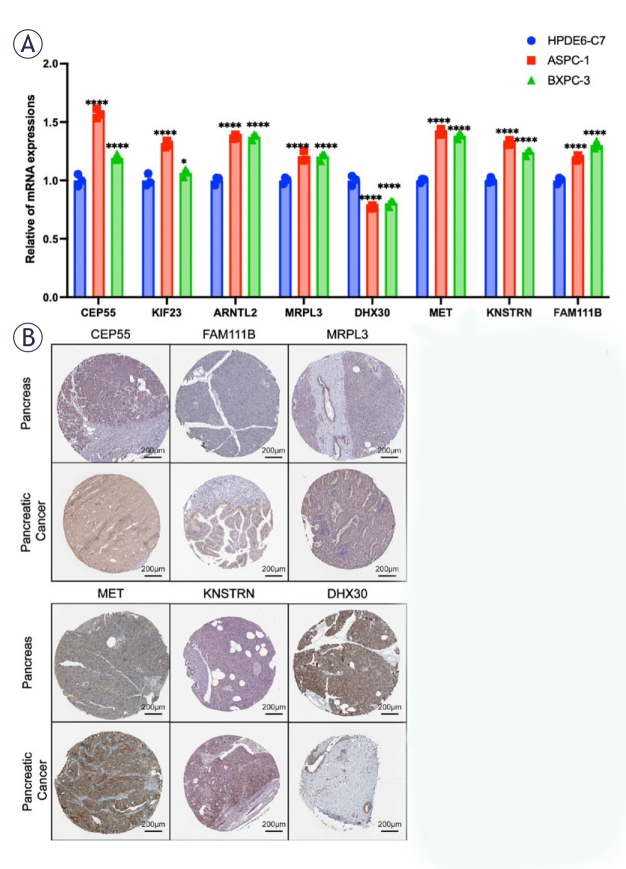
Cell experiment and screening of low cuproptosis-related genes (CRGs). **(A)** quantitative real time-PCR (qRT-PCR) to assess the expression of 8 cuproptosis-related genes (CRGs) in pancreatic epithelial cells (HPDE6-C7) and two pancreatic cancer cell lines (ASPC-1 and BXPC-3). **(B)** Immunohistochemical analysis revealed elevated protein expression of *CEP55, FAM111B, MRPL3, MET*, and *KNSTRN* in pancreatic cancer tissues, while *DHX30* exhibited significantly higher expression in normal pancreatic tissues than in pancreatic cancer tissues among the 8 CRGs.

### Cellular localization and prognostic analysis of 8 hub genes

To investigate the expression patterns of hub genes across distinct cell types, we conducted a detailed single-gene analysis focusing on the eight identified hub genes (Figure 5A–H). The results, revealed that *CEP55, KIF23, ARNTL2, MRPL3* and *MET* displayed prominent expression in Epithelial cells. Furthermore, *FAM111B, DHX30* and *KNSTRN* demonstrated a prevalent expression pattern in all cell types.

Subsequently, a comprehensive prognostic analysis was performed for these eight genes. The findings, depicted in Figure 5I–P, unveiled that, with the exception of *DHX30*, PAAD patients with elevated expression levels of the remaining seven genes experienced significantly worse prognoses compared to those with lower expression levels. These results underscore the potential prognostic significance of these genes in the context of PAAD, shedding light on their differential expression across various cell types.

### Confirming the expression levels of CRGs

The expression of CRGs in pancreatic epithelial cells (HPDE6-C7) and two pancreatic cancer cells (ASPC-1, BXPC-3) was detected by RT-qPCR. The results showed that except for *DHX30*, mRNA expression levels of other 7 genes in cancer cells were higher than those in normal cells (Figure 6A). Immunohistochemical results showed that among the 8 CRGs: *CEP55, FAM111B, MRPL3, MET*, and *KNSTRN* had higher protein expression in pancreatic cancer tissues, while on the contrary, the protein expression of *DHX30* in normal pancreas was significantly higher than that in pancreatic cancer tissues (Figure 6B). These results were also consistent with the above RT-qPCR results. In addition, *KIF23* was strongly positive in normal pancreatic tissue and PAAD, and the difference between them could not be significantly distinguished. No *ARNTL2* positive signal was detected in the immunohistochemical results of pancreas and pancreatic cancer. Therefore, the immunohistochemical results of the above two genes were not shown.

### CEP55 promotes cell proliferation and invasion in ASPC1 cells

**FIGURE 7.**
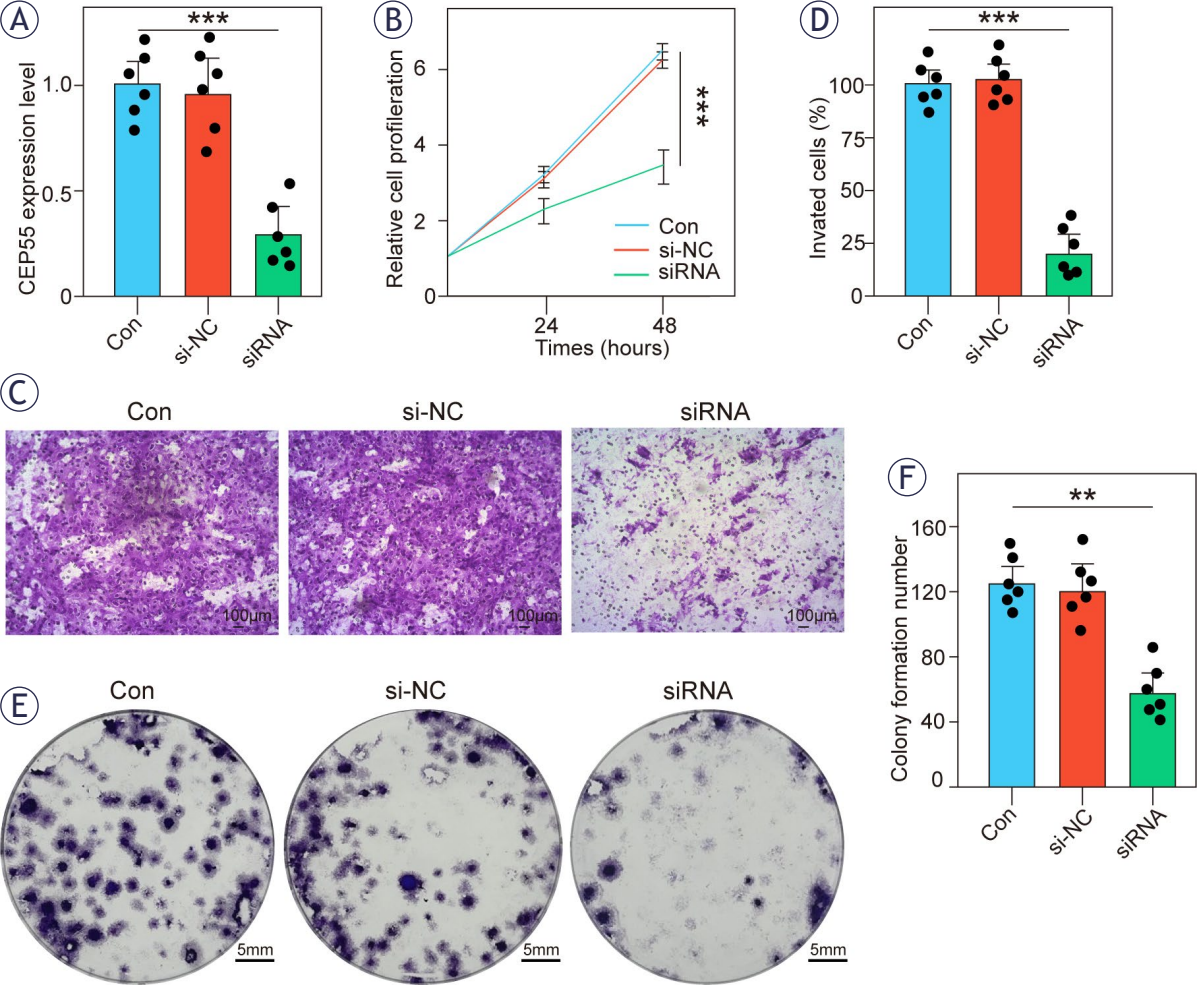
*CEP55* knockdown inhibits pancreatic adenocarcinoma (PAAD) in ASPC1 cell line. **(A)** quantitative real time-PCR (qRT-PCR) to assess the expression of *CEP55*. **(B)** The results of Cell Counting Kit-8 (CCK8). **(C-D)** *CEP55* knockdown inhibits cell invasive ability by transwell assay. **(E-F)** *CEP55* knockdown inhibits colony formation in ASPC1 cell line.

To investigate the functional role of *CEP55* in PAAD progression, we successfully knockdown *CEP55* in the ASPC1 cell line using siRNA silencing. While siRNA silencing achieved only partial reduction of *CEP55* expression (Figure 7A), this partial suppression was sufficient to markedly impair cell viability (Figure 7B), migration, and invasion (Figure 7C–D), as well as colony formation (Figure 7E–F) compared to control groups. These findings suggest that the downregulation of *CEP55* effectively inhibits cell proliferation, migration, invasion, and colony formation, even under incomplete silencing conditions. Notably, siRNA provides rapid and transient gene suppression, whereas complete and sustained knockdown methods may yield more pronounced phenotypic effects. Nevertheless, our data underscore that partial *CEP55* downregulation significantly attenuates key hallmarks of PAAD progression.

## Discussion

Pancreatic adenocarcinoma (PAAD) is a malignancy with a very poor prognosis, and the 5-year survival rate for this disease is statistically less than 7%.^1,24^ In the past, to prolong the survival of PAAD patients, we could only use surgical and chemotherapeutic treatments, although their effectiveness was in fact limited.^25,26^ In recent years, with the development of cancer immunotherapy in full swing^27,28^, more and more scholars have also started to put their eyes on the relationship between PAAD and immunotherapy.^29,30^ For example, Fengjiao Li *et al*. found that Glucose transporter 1 (GLUT1) regulates the tumor IME through an ncRNA-mediated network and promotes PAAD tumor metastasis^31^; However, up to now, immunotherapy has not yielded satisfactory results in PAAD.^7,32^ This makes the research in immunotherapy of PAAD full of potential. Cuproptosis, a newly discovered form of cell death, has a great role in the tumor microenvironment^16,33,34^ and has been extensively studied in the field of cancer.^35,36^ However, there are still few studies on cuproptosis in PAAD. The aim of this study was to investigate the prognostic value of cuproptosis in PAAD and its role in the IME using multi-omics techniques such as transcriptomic analysis and single-cell analysis.

In this study, through an extensive analysis of PAAD data from the TCGA and GEO databases, we divided PAAD patients into high-risk and low-risk groups based on the subsequently calculated cuproptosis scores. The results showed that both in the TCGA and GEO cohorts, the high-risk group showed a poorer outcome. Since there are no studies to date on the association between CRG and the occurrence of PAAD^37^, we constructed an 8-gene model related to CRGs score based on differential expression analysis and WGCNA results. In addition, by using ROC curves, we found that the model also showed high accuracy in assessing the prognosis of PAAD patients at 2, 3 and 5 years. The results of the immune microenvironment and mutation correlation analysis showed similarity in mutated genes between high and low risk groups, while there were some differences in the mutation rates of the same genes. We identified *CEP55* as the hub gene with the greatest difference through qPCR and immunohistochemistry. In previous studies of *CEP55* in breast cancer, *CEP55* knockdown significantly reduced cell survival, proliferation, and migration.^38^ In this study, *CEP55* knockdown effectively inhibited cell proliferation, migration, invasion and colony formation, demonstrating that *CEP55* can promote the progression of PAAD. Moreover, we used single-cell data to explore the distribution of 10 CRGs genes in different types of PAAD cells. In addition, the distribution of 8 CRGs in different cell types also had mutual corroboration with the results of immune infiltration analysis based on CRGs scores, further exploring the possible mechanism of cuproptosis in PAAD.

Despite the rapid development of cancer immunotherapy, the application of this approach in PAAD has had little success for a long time^39^, which is one of the reasons why the prognosis of PAAD is so poor. The TME, also known as the stromal compartment, is composed of cancer-associated fibroblasts (CAFS) with immune cells.^40^ The prevailing view is that the TME can activate and transform growth factor beta to drive the recruitment of immunosuppressive cells, thereby limiting immune cell infiltration and impairing their function in the tumor^41^, while the abundant stromal component in the tumor is one of the specific features of PAAD.^42,43^ Therefore, PAAD was considered to be low immunogenic.^44^ In recent years, however, new breakthrough points have been made in this thorny historical problem. A new model has been used to convert non-immunogenic PAAD into immunomodulatory immunogenic lesions^45^, while tertiary lymphoid structures (TLS) in the tumors of some PAAD patients have been identified to contribute to antitumor immunity.^46^ The role of various immunomarkers in PAAD has been extensively studied.^47–49^ It is important to understand the TME of PAAD based on new perspectives. In the present study, we found that immune-related processes play a crucial role in PAAD by differential expression analysis and enrichment analysis. Therefore, we utilized seven algorithms to explore differences in the cancer immune microenvironment. The results showed that the infiltration level of immune cells in the high CRGs group was significantly lower than that in the low CRGs group, and the statistically significant immune cells were also consistent with the pathway obtained by enrichment analysis. Thus, the high CRGs group may be more likely to benefit from immunotherapy.

*CEP55* is a key protein in cytokinesis^50^, whose overexpression is associated with genomic instability, one of the hallmarks of cancer.^51^*CEP55* overexpression promotes genomic instability by^51^, activates PI3k/Akt pathway signaling^52^ and inhibition of apoptosis.^53^ However, no previous studies have investigated its role in cuproptosis. *CEP55* is a gene involved in lipid metabolism^54^, and lipoacylation is a mitochondrial process essential for cuproptosis^55^, so we hypothesized that knocking down *CEP55* could reduce the efficiency of lipoacylation and thus inhibit cuproptosis. However, in our study, knocking down *CEP55* inhibited the progression of PAAD, so *CEP55*’s tumor-promoting effect may outweigh its effect of enhancing cuproptosis to inhibit PAAD.

While our multi-omics analyses and in vitro experiments have established *CEP55* as a critical oncogene in PAAD, this study has certain limitations. First, transient knockdown in siRNA-dependent cell lines, while valid for initial validation, does not fully generalize the sustained gene suppression achievable by the complete knockdown approach. Second, While *CEP55* has been identified and functionally assessed, its broader biological function has not been fully validated. Therefore, further validation of *CEP55* in vivo experiments is needed.

## Conclusions

This study constructed a cuproptosis-related prognostic model for PAAD through multi-omics techniques. Moreover, the cancer immune microenvironment and tumor mutational burden of two CRGs groups were assessed. Finally, *CEP55* was identified as the hub gene of PAAD in this study. We verified the biological function of *CEP55* in vitro. This provides a new potential therapeutic target for PAAD.

## Supplementary Material

Supplementary Material Details
