# Supplementary material for: Cuproptosis-related gene CEP55 as a biomarker of pancreatic adenocarcinoma via multi-omics techniques and experimental validation
Source: Radiol Oncol. 2025 Jul 18;59(3):368–82. doi: 10.2478/raon-2025-0042 (PMC12441886; doi:10.2478/raon-2025-0042)
Supplement: Supplementary file 1 — Supplementary Material Details [file raon-2025-0042_sm.pdf]

Cuproptosis-related gene *CEP55* as a biomarker of pancreatic adenocarcinoma via multi-omics techniques and experimental validation

Riyuan Zhang, Zixia Xu, Yurui Zhuang, Yuzhe Shi, Ziyi Guo, Chong Chen

doi: 10.2478/raon-2025-0042

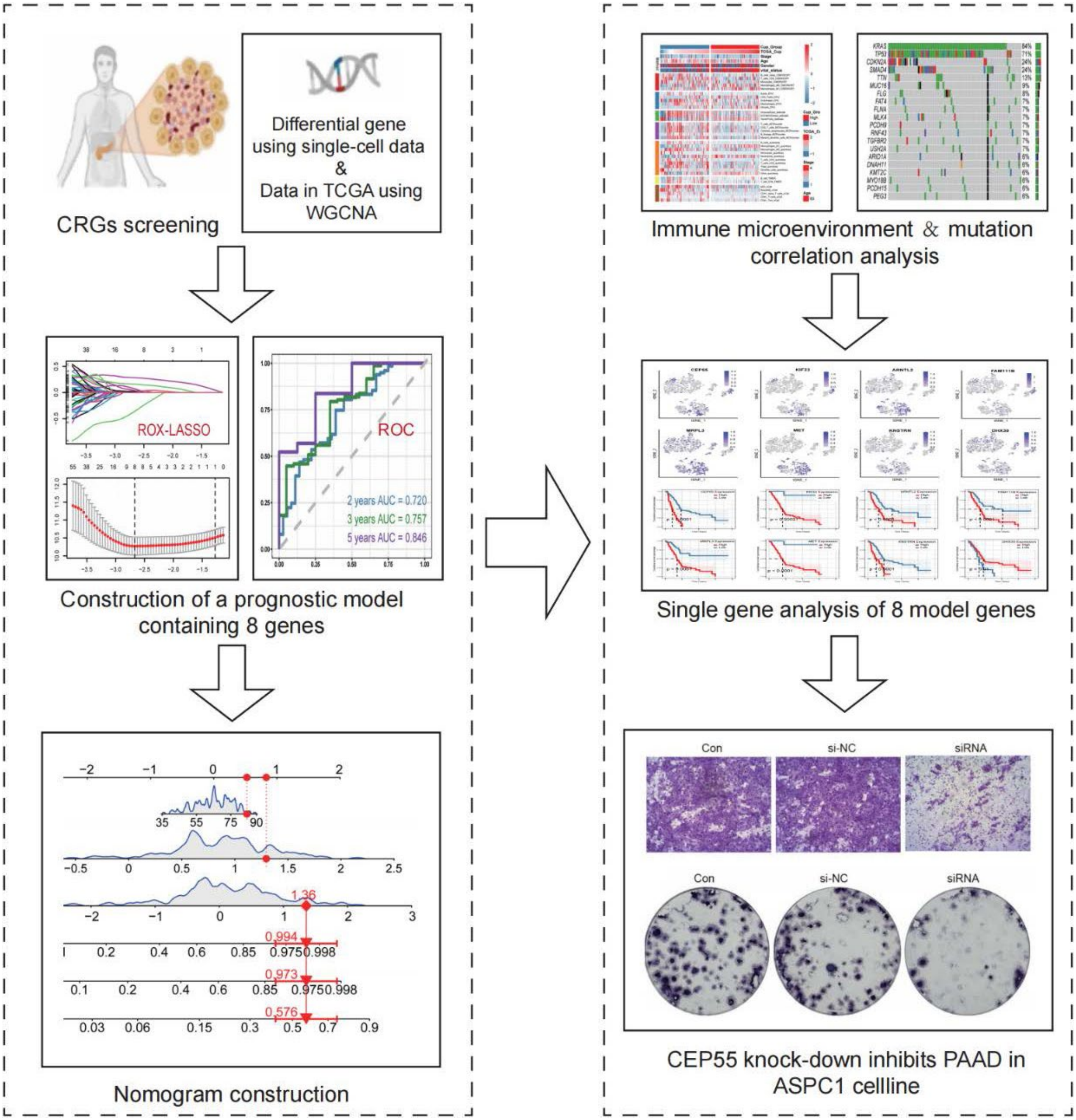

SUPPLEMENTARY FIGURE 1. Flow chart.

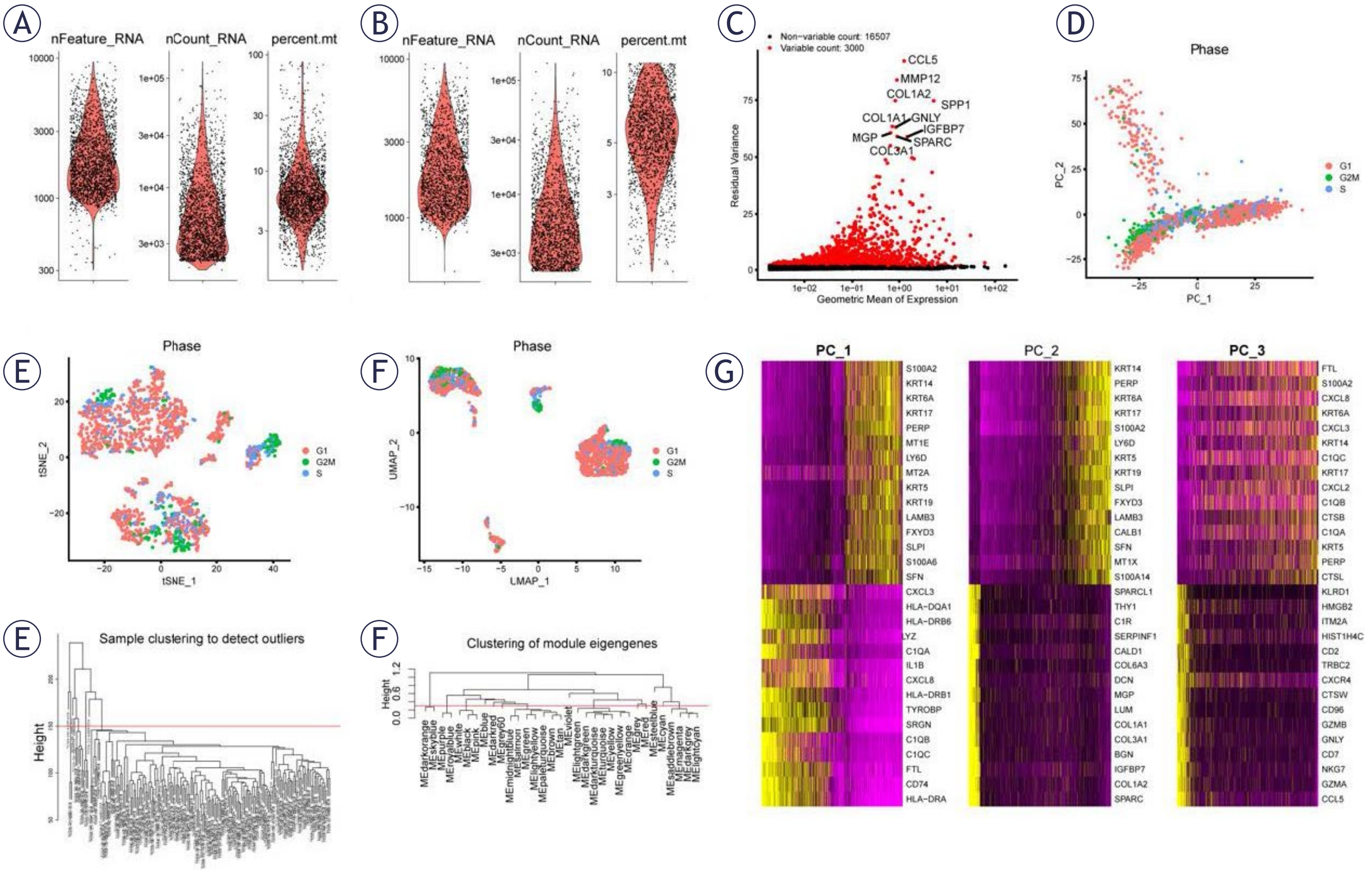

SUPPLEMENTARY FIGURE 2. Single-cell analysis quality control process. **(A-F)** The quality control process of the single-cell analysis.

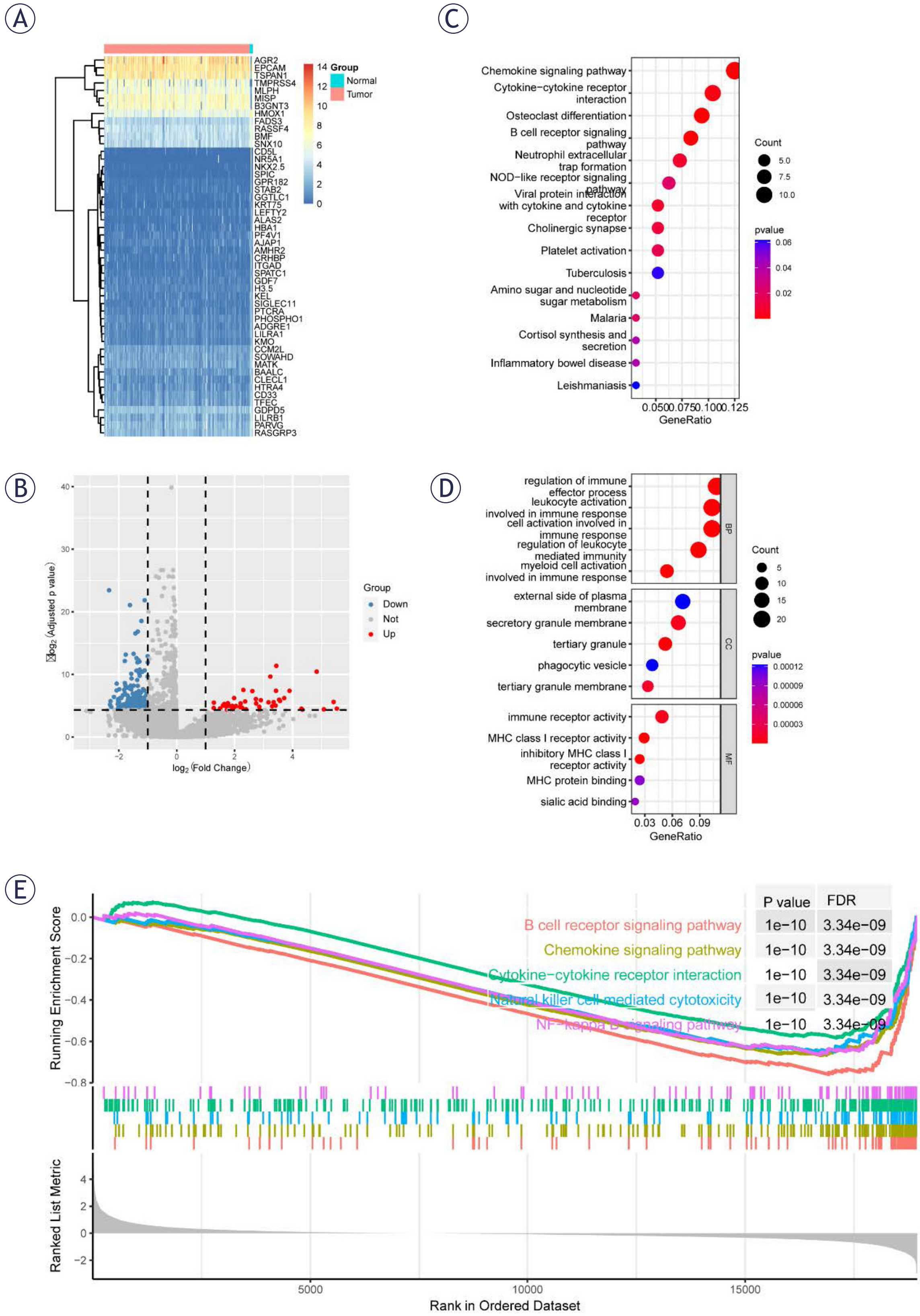

SUPPLEMENTARY FIGURE 3. Differential expression analysis and enrichment analysis. **(A, B)** Differential expression analysis. **(C-E)** Enrichment analysis. The identification of key pathways, such as chemokine signaling, cytokine-cytokine receptor interaction, and B cell receptor signaling, underscores the pivotal role of immune-related processes in PAAD

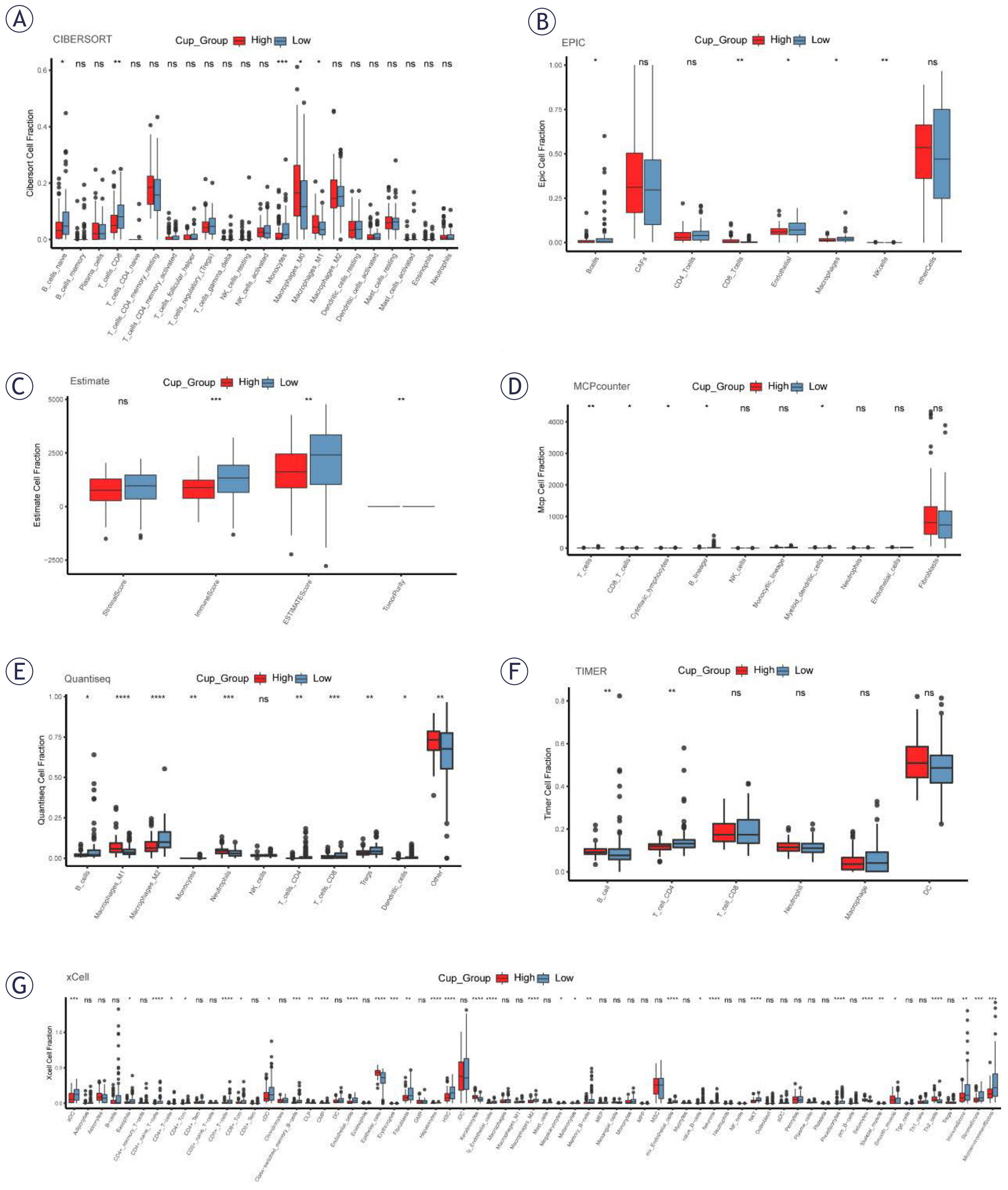

SUPPLEMENTARY FIGURE 4. Statistical analysis from 7 immune infiltration algorithms. **(A-G)** The results reveal higher immune cell infiltration in the low CPPS group, primarily involving T cells, B cells, and macrophages.

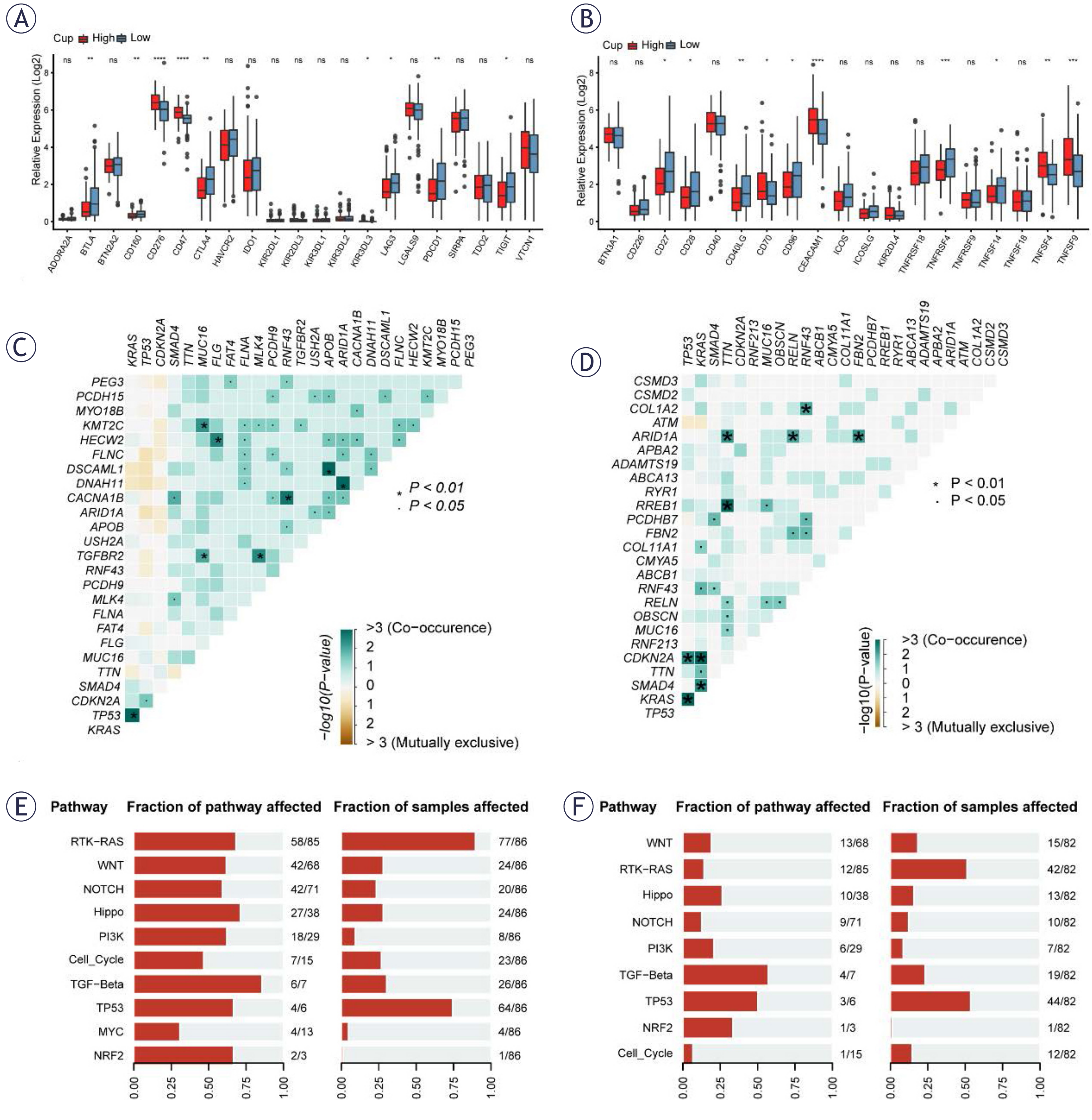

SUPPLEMENTARY FIGURE 5. The results of immune infiltration analysis and tumor mutation analysis. **(A, B)** Differential expression of immune checkpoint-related genes. **(C, D)** Analysis of the correlation of mutated genes. **(E, F)** Analysis results of pathways related to mutated genes. The results indicate mutations in key pathways, encompassing RTK-RAS, WNY, TGF-Beta, and TP53.

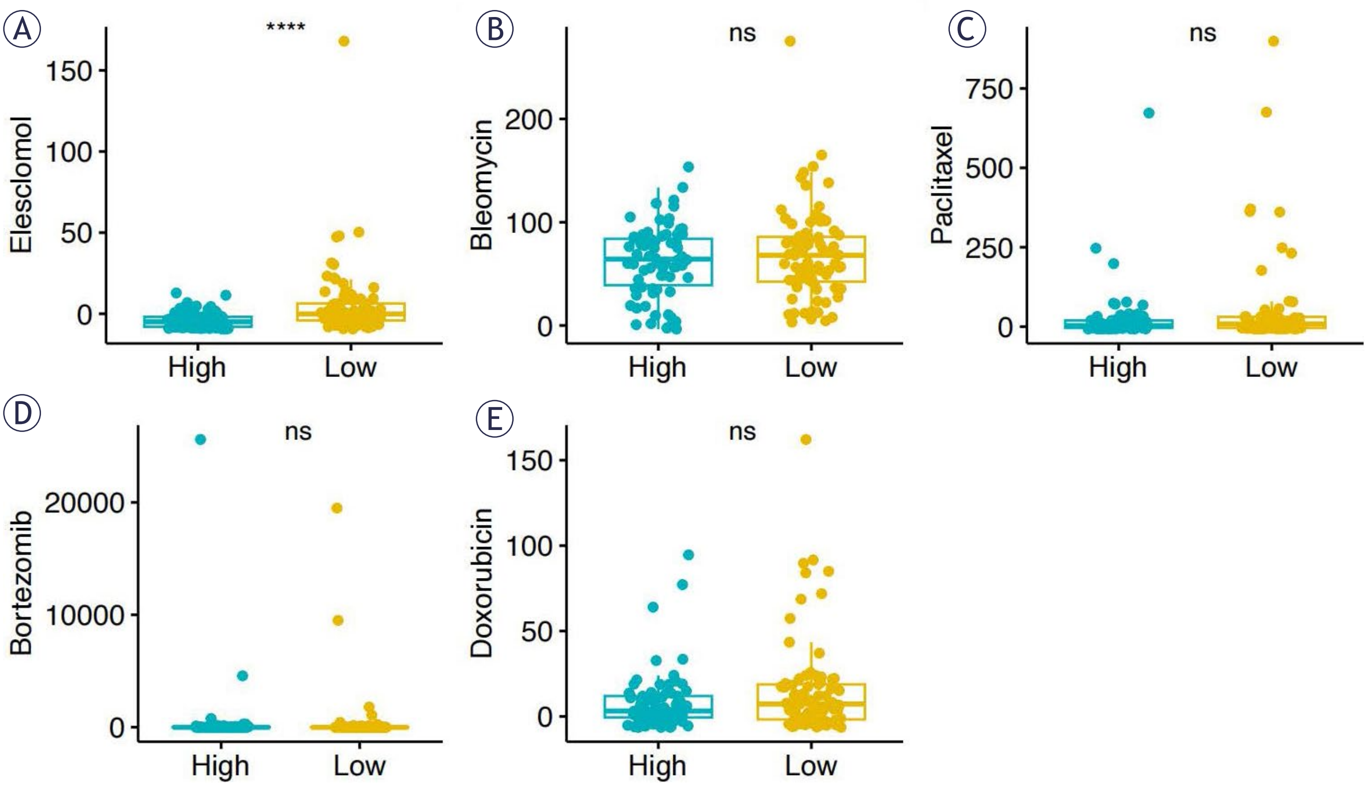

SUPPLEMENTARY FIGURE 6. Drug sensitivity between high and low cuproptosis-related genes groups. **(A-E)** IC50 of five copper metabolism-related drugs in two CRGs group.
